# Exploring the effects of exercise on T cell function and metabolism in cancer: a scoping review protocol

**DOI:** 10.3389/fphys.2025.1655306

**Published:** 2025-08-29

**Authors:** J. L. Low, H. J. Lee, B. A. Edgett, K. Romme, S. N. Culos-Reed, J. B. Lee

**Affiliations:** ^1^ Riddell Centre for Cancer Immunotherapy, Arnie Charbonneau Cancer Institute, Cumming School of Medicine, University of Calgary, Calgary, AB, Canada; ^2^ Faculty of Kinesiology, University of Calgary, Calgary, AB, Canada; ^3^ Health Sciences Library, Memorial University of Newfoundland, St. John’s, NL, Canada; ^4^ Department of Oncology, Cumming School of Medicine, University of Calgary, Calgary, AB, Canada; ^5^ Department of Psychosocial Resources, AE Child Comprehensive Cancer Centre, Cancer Care, Alberta Health Services, Calgary, AB, Canada; ^6^ Department of Biochemistry and Molecular Biology, Cumming School of Medicine, University of Calgary, Calgary, AB, Canada; ^7^ Department of Microbiology, Immunology, and Infectious Disease, Cumming School of Medicine, University of Calgary, Calgary, AB, Canada

**Keywords:** cancer, immunotherapy, exercise, t cells, immune cells

## Abstract

**Background:**

The global burden of cancer is escalating, and improved strategies for disease prevention and treatment are needed. The immune system, particularly T cells, plays a crucial role in cancer surveillance and eradication. Immunotherapy strategies that leverage the anti-cancer T cell response have significantly advanced therapeutic approaches to cancer treatment. Exercise is a lifestyle factor that naturally stimulates and strengthens the immune system. This interaction may not only be linked to the benefits of exercise in decreasing cancer risk and increasing survival but may also have the potential to be harnessed to enhance current forms of immunotherapy. Central to the exercise-immune system axis and anti-cancer control are T cells, yet little is known about how exercise might influence their function and metabolic fitness.

**Objective:**

We propose a scoping review with the aim to understand and summarize the current literature on the effects of exercise on T cell function and metabolism in cancer, identifying potential key mechanisms, impacts on therapeutic applications, exercise modalities, and associated outcomes.

**Methods:**

This scoping review will be conducted according to the methodology for scoping reviews laid out by JBI. The Preferred Reporting Items for Systematic Reviews and Meta-Analysis Extension for Scoping Reviews (PRISMA-ScR) will be followed. Experimental studies involving i) humans OR mammals and ii) examinations of T cell function and metabolism, and iii) exercise interventions, and iv) in the context of cancer will be included.

**Results:**

Data search, screening and extraction will take place from June 2025-December 2025. Preliminary searches conducted while developing the initial search strategy resulted in an estimated ∼700–1000 titles and abstracts for initial screening.

**Conclusion/Implications/Dissemination:**

The proposed scoping review will be submitted for publication upon completion. The potential findings hold profound implications for future research in this field, providing mechanistic insights into the exercise-immune system axis that can be leveraged to enhance immune-based approaches for cancer prevention, treatment, and long-term survivorship.

## Introduction

### Exercise as an adjunct to cancer care

The global burden of cancer is escalating, with 20 million new cases and 10 million deaths (one in six of all deaths) reported in 2022 (Bray et al., 2024). In the context of growing aging demographics worldwide (Garmany et al., 2021) and the consistent association between age, cancer risk (Laconi et al., 2020), and immunosenescence (Liu et al., 2023), this alarming trend highlights the need for effective preventative and therapeutic measures in the fight against cancer, with a particular focus on older adults. Exercise has garnered significant attention within the research and clinical communities, demonstrating a myriad of health benefits across the cancer continuum, including mitigated cancer risk (Matthews et al., 2019; Patel et al., 2019), improved quality of life (Gerritsen and Vincent, 2016), decreased chance of recurrence (Cormie et al., 2017; Morishita et al., 2020), and increased survival (Cormie et al., 2017; Lavery et al., 2023; Courneya et al., 2025). In addition, exercise is well-known to induce favourable adaptations in whole-body physiology and metabolism (McGee and Hargreaves, 2020), with preclinical studies discovering that these benefits could be harnessed to enhance anti-cancer therapeutics (Ashcraft et al., 2019; Yang et al., 2021; Kurz et al., 2022; Phelps et al., 2025).

Recently, a multi-centered randomized controlled trial involving 889 colon cancer patients provided compelling evidence that structured exercise after adjuvant chemotherapy improves disease-free and long-term survival (Courneya et al., 2025). While biological mechanisms underpinning this outcome remain elusive, exercise elicits a systemic response impacting multiple organ systems, including the immune system (which is highly responsive) (Chow et al., 2022; Fiuza-Luces et al., 2024). Given the crucial role of the immune system in combatting cancer (Chen and Mellman, 2013), the exercise-immune system axis likely contributes, at least in part, to the observed effects of exercise on cancer risk, recurrence, and survival benefits in individuals living with and beyond cancer.

### The exercise-immune system axis: focus on T cells

Immune cell mobilization into tumours is commonly associated with better prognosis (Karn et al., 2017). Exercise effectively mobilizes cytotoxic immune cells, such as natural killer (NK) cells and CD8^+^ T cells, into the circulation in both healthy individuals and cancer patients (Schauer et al., 2022; Djurhuus et al., 2023; Koivula et al., 2023). Although the downstream actions of exercise-mobilized immune cells in patients are not yet fully understood, early transcriptomic analysis provides some insight. In one study, newly diagnosed breast cancer patients were randomized to a prehabilitation exercise intervention combining aerobic and resistance exercise or a mind-body psychological support control group (Ligibel et al., 2019). From study enrolment to surgery (about 30 days), the patients in the exercise group were engaged in two supervised 60–90-minute sessions per week and unsupervised aerobic exercise on their own, accumulating 10-times the amount of weekly exercise compared to the control group. The tumour samples were compared between the groups at baseline and after surgical excision. Enrichment of proinflammatory transcriptional pathways such as cytokine-cytokine receptor interaction, NK cell–mediated cytotoxicity, and T cell receptor signaling, was seen in samples collected from the exercise group, suggesting that exercise can promote an inflammatory milieu within the tumour microenvironment (TME) conducive to immune-mediated anti-cancer control.

Central to the exercise-immune system axis and anti-cancer control are T cells, which can recognize and eliminate cancer cells by binding to antigen peptides presented on the major histocompatibility complex (MHC) of malignant cells (Waldman et al., 2020). Identified by their canonical surface marker CD3, different T cell subsets exist based on the expression of either CD4 or CD8 co-receptors, namely CD4^+^ T cells and CD8^+^ T cells, respectively. CD8^+^ T cells are known as cytotoxic effector cells that directly target and destroy cancer cells. Their abundance and functional status within tumours have been correlated with better responses to immune-based treatments and improved patient outcomes (Wang et al., 2024). Additionally, CD4^+^ T helper cells contribute to anti-cancer immunity by enhancing the activity of CD8^+^ T cells and orchestrating the overall immune response through the production of signaling molecules called cytokines (Tay et al., 2021). T regulatory (T_reg_) cells are a subset of CD4^+^ T cells that express the forkhead box P3 (Foxp3) transcription factor, promoting immune tolerance and preventing autoimmune reactions; however, their role can have paradoxical implications in cancer. For example, in the tumour, T_reg_ cells can suppress CD8^+^ T cell activity, thereby fostering an immunosuppressive environment that facilitates tumour progression and immune evasion associated with poor prognosis in various cancers (Wang, 2008; Facciabene et al., 2012; Hu et al., 2021; Liu et al., 2024).

### Preclinical insights: exercise enhances T cell anti-cancer function

Preclinical evidence supports exercise as a potent stimulus that could enhance T cell-mediated anti-cancer immunity. Regular aerobic exercise provides protective effects against pancreatic ductal adenocarcinoma (PDAC) in mice through an intricate crosstalk between the exercising muscle (Hsueh et al., 2022) and CD8^+^ T cells. Specifically, exercised muscle releases interleukin-15 (IL-15), a cytokine that promotes T cell cytotoxic capacity (Klebanoff et al., 2004; Lee et al., 2024) and long-term survival (Rochman et al., 2009; Sindaco et al., 2023), which interacts with CD8^+^ T cells expressing its cognate receptor IL-15Rα (Kurz et al., 2022). Despite poor CD8^+^ T cell infiltration characteristic of PDAC (Bear et al., 2020), exercise mobilized and trafficked IL15Rα^+^ CD8^+^ T cells into the tumour, and transcriptomic analysis using single-cell RNA sequencing of tumour samples from the exercised mice showed enrichment of effector CD8^+^ T cells with heightened cytotoxic signaling. In murine breast cancer models, aerobic exercise delayed tumour growth by increasing CXCR3-dependent signaling that promotes T cell infiltration to tumours. Notably, these exercise-induced benefits were abrogated by CD8^+^ T cell depletion as well as in CXCR3 genetic knockout models (Gomes-Santos et al., 2021). Similar findings were observed in all conditions of mice performing aerobic exercise training before, after, or before and after Lewis lung cancer inoculation (Miao et al., 2024). In a separate study, exercise prior to breast tumour inoculation slowed its growth, which was associated with a significant decrease in the proportion of immunosuppressive T_reg_ population in the tumour (Hagar et al., 2019). Together, these findings underscore the impact of exercise on skewing T cell dynamics towards more potent anti-cancer immunity towards more potent anti-cancer immunity.

Immunotherapies leveraging the T cell’s ability to recognize and elicit targeted killing of malignant cells have significantly advanced therapeutic approaches to cancer treatment. The leading form of immunotherapy is adoptive cellular therapy (ACT), such as Chimeric Antigen Receptor-T cell (CAR-T cell) therapy. Current CAR-T cell therapy involves extracting the patient’s T cells, enhancing their anti-cancer properties in the lab, and infusing them into the patient (Huang et al., 2020). While ACT has achieved impressive clinical outcomes in patients with limited treatment options (Yang and Rosenberg, 2016), patients’ T cells are often compromised due to various factors, such as constant encountering of cancer cells and/or metabolic deprivation in the TME, resulting in T cell exhaustion, a hallmark of malfunctioning T cells. This ultimately limits T cells’ ability to mediate durable anti-cancer effects in patients and as ACT products. In a novel way, there is potential to harness the performance-enhancing effects of exercise on T cells to overcome current challenges in ACT and improve its efficacy.

Skeletal muscles are secretory organs that produce a milieu of signaling molecules with autocrine, paracrine, and endocrine functions (Chow et al., 2022). Specifically, the exercising muscle produces cytokines (or ‘myokines’) and metabolites that are intriguingly common to T cell biology and immunometabolism. Indeed, despite two seemingly distinct fields of study, muscle-derived cytokines and metabolites have been shown to impact T cell function. During chronic viral infection, muscle-derived IL-15 reduces CD8^+^ T cell exhaustion and increases T cell factor (Tcf1) protein expression, which is crucial for maintaining T cell stemness associated with long-term persistence and proliferative potential (Wu et al., 2020). In contrast, muscle-specific deletion of IL-15 impaired CD8^+^ T cell function, upregulating exhaustion markers such as PD-1, LAG-3, and TIGIT, while downregulating cytotoxic effector molecules such as IFNγ, TNFα, and granzymes. Importantly, less differentiated stem-like memory T cells have been associated with more durable and superior clinical outcomes in patients treated with T cell-based therapies (Fraietta et al., 2018), and IL-15 has been used in CAR-T cell manufacturing to preserve the stem-cell memory phenotype (Alizadeh et al., 2019).

### Implications for immunotherapy: potential of exercise to enhance adoptive T cell therapy

Adoptive transfer of T cells obtained from exercised mice yields superior anti-tumoural effects *in vivo*, indicating that exercise induces changes in T cells that are favourable for better ACT outcomes (Rundqvist et al., 2020). From a metabolic perspective, muscle-T cell crosstalk may occur through exercise-derived metabolites. Lactate, primarily produced by muscle during exercise, is one such example. CD8^+^ T cells treated with lactate enhance their effector profile (Rundqvist et al., 2020). Furthermore, lactate has been shown to increase CD8^+^ T cell stemness by increasing Tcf7 gene expression, which encodes the transcription factor Tcf1, associated with T cell memory and stemness (Feng et al., 2022). Within the TME, unmatched glucose consumption by cancer cells restricts the T cell’s ability to fuel its effector function, leading to tumour progression (Chang et al., 2015). To address this issue, T cells that can metabolically compete in the TME are needed. In one study, aerobic exercise training provided protective effects against colon cancer in mice, with improvements observed in the mitochondrial content and function of tumour-infiltrating CD8^+^ T cells (Voltarelli et al., 2024).

These findings on the molecular network between exercise and T cells highlight the potential of exercise-derived factors in inducing favourable adaptations in T cell function, inspiring novel pathways that could be exploited to enhance the effectiveness of T cell-based cancer therapy. For example, prehabilitation exercise interventions prior to T cell extraction for ACT could yield more functional T cells used for product manufacturing or, possibly, regular exercise post-infusion may provide metabolites and myokines to support more durable anti-tumoural activity of T cells. In cases where implementing exercise is challenging, such as in high-risk patients, the immune-boosting effects of exercise could be indirectly harnessed in the context of allogeneic ACT, which uses healthy donor-derived immune cells for treatment.

### Rationale for this review

Building on this understanding of T cells and their role in the fight against cancer, it is important to further explore how exercise might influence their function and behavior—particularly in light of the growing need for effective preventative and therapeutic strategies in cancer. Many of the existing reviews on exercise and T cells in cancer have primarily focused on cell frequency and cytokine production, often overlooking the functional and mechanistic changes that occur within T cells. This limited focus has impeded a deeper understanding of how exercise may influence T cell function and its potential role in cancer immunotherapy. In light of this, there is a need for scoping reviews to synthesize existing knowledge on the mechanisms, types of exercise, and outcomes related to T cell function and metabolism in the context of cancer, thus providing a foundation for future research and clinical practice. Elucidating these connections holds promise for informing the development of tailored exercise interventions that aim to optimize anti-cancer immunity and discover novel strategies to enhance cancer immunotherapy, thereby leading to improved overall patient outcomes.

### Objective

**FIGURE 1:**
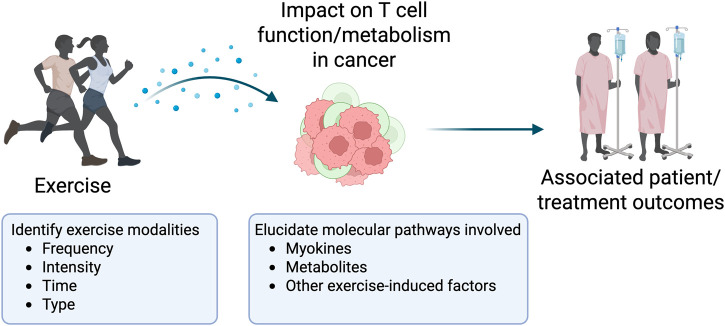
Visual representation of the proposed scoping review aim. Created in BioRender. Lee, J. (2025) https://BioRender.com/bn3b3x6.

We propose a scoping review with the aim to understand and summarize the current literature on the effects of exercise on T cell function and metabolism in cancer, identifying potential key mechanisms, exercise modalities, and associated outcomes (Figure 1).

## Methods and analysis

### Scoping review design

This scoping review will be conducted according to the methodology for scoping reviews laid out by JBI(Arksey and O’Malley, 2005). The Preferred Reporting Items for Systematic Reviews and Meta-Analysis Extension for Scoping Reviews (PRISMA-ScR) will be followed (McGowan et al., 2020).

### Research questions

The primary research question of the proposed scoping review is: Does exercise impact T cell function and metabolism in cancer? Secondary questions are: What molecular pathways are implicated in T cell metabolism during and after exercise? Are there specific markers or metabolic shifts in T cells linked to exercise? How does exercise influence T cell functions (activation, proliferation, and differentiation) through metabolic changes? What are the effects of either acute or chronic exercise training on T cell metabolism? Do different types of exercise (aerobic versus intervals versus strength training) differ in their impact on T cell function?

### Protocol registration

The finalized, published protocol will be freely available and registered on the Open Science Framework (OSF) website.

### Population, concept, context

The inclusion criteria for the proposed scoping review have been defined according to Chapter 10 of the JBI Manual for Evidence Synthesis (Aromataris E et al., 2024).

#### Population

This review will include studies involving individuals of all ages with cancer or animal models with cancer. Both human (*in vivo*) and non-human (*in vitro*) studies will be considered to capture a comprehensive understanding of the exercise-induced effects on T cells across different biological systems and cancer types.

#### Concept

The central concept of this review is the effect of exercise on T cells within the context of cancer. This includes studies examining how various forms, intensities, and durations of exercise influence T cell metabolic function, phenotype, activation status, infiltration into the tumour microenvironment, or functional anti-tumour activity. We have chosen to include low-intensity exercise modalities (e.g., yoga and tai chi) as these gentler forms of exercise have demonstrated significant physical improvements in those with disease impairments (Desveaux et al., 2015). Emerging data suggest that low-intensity exercise can modulate key regulators of T cell metabolic fitness, which in turn, may contribute to anti-tumour immunity in cancer patients (Venkatesh et al., 2020; Estevao, 2022; Kaushik et al., 2022). To address the differential impact of low-intensity versus moderate- or high-intensity activities on immune cell function, the results from low-intensity exercise will be categorized differently from moderate- or high- intensity exercise.

#### Context

**FIGURE 2:**
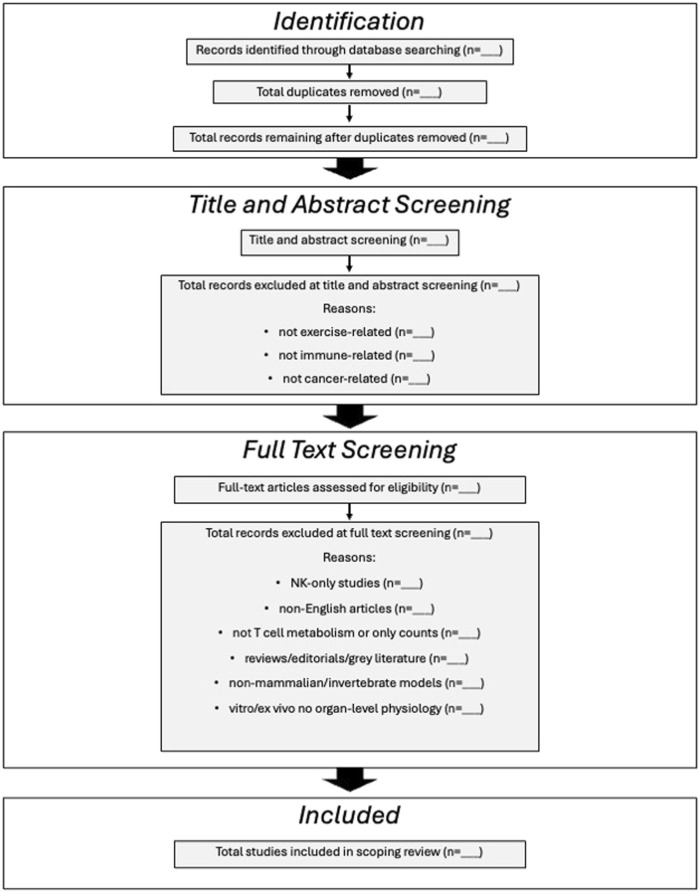
Sample template for resultant reporting on screening and exclusions.

All experimental study types (randomized controlled trials, observational studies, cohort studies) will be included. Studies from clinical, preclinical, laboratory, or community-based settings will be included to ensure comprehensive coverage of the available evidence. Studies will be excluded from this scoping review if they are not related to exercise or immune function, or if they do not focus on cancer. Studies that only report T cell counts without providing mechanistic or functional insights will also be excluded. Mechanistic and functional insights are defined as quantitative readouts of T cell effector function or metabolic activity, and will include cytokine secretion (e.g., ELISA or intracellular staining), cytotoxicity assays, immunophenotype, metabolic flux analyses (e.g., Seahorse/Oroboros measurements of glycolysis and oxidative phosphorylation), and omics-based profiling, such as metabolomics, transcriptomics, and proteomics. Additionally, studies that focus solely on NK cells and do not examine T cells will be excluded. Review articles, editorials, opinion pieces, and grey literature will be excluded, though they may be flagged or tagged for reference trailing. We will include studies in mammalian species whose physiology and disease-relevant mechanisms closely parallel those in humans, specifically, non-human primates, rodents (including transgenic cancer models), canines, and porcine models. Studies involving non-mammalian species (e.g., fish, amphibians, reptiles), invertebrates, and *in vitro* or *ex vivo* systems lacking intact organ-level physiology will also be excluded. Finally, articles published in languages other than English will be excluded. See Figure 2 for a detailed tracking template.

### Information sources and databases

A comprehensive literature search will be conducted using the following electronic databases: Ovid MEDLINE, EMBASE, Web of Science and SPORTDiscus. These databases have been selected to capture a broad range of biomedical, exercise science, and interdisciplinary research relevant to the review topic. The reference lists of included studies and relevant reviews will also be hand-searched to identify any additional studies not captured through the database searches. See Figure 2 for details.

### Citation management and data collation

All identified citations will be organized and managed using the online software Covidence (Veritas Health Innovation, Melbourne, Australia). Covidence is a web-based collaboration software platform that streamlines the production of systematic and other literature reviews. References will be handled through Mendeley Cite (Elsevier, Cambridge, Massachusetts). A number of independent reviewers will screen titles and abstracts to assess their relevance based on the inclusion criteria. Studies deemed relevant will proceed to full-text assessment, again conducted by a number of independent reviewers. Eligible studies will then undergo data extraction. Studies that do not meet the inclusion criteria will have the reasons for exclusion documented and reported in the scoping review. Any disagreements during the title-abstract or full-text screening phases will be resolved by a third independent reviewer. The outcomes of the search and study inclusion process will be fully reported in the final scoping review, with results presented in a PRISMA-ScR flow diagram (McGowan et al., 2020).

### Methodological appraisal

Given that this is a scoping review, formal methodological quality appraisal of included studies will not be conducted. The purpose of this review is to map the available evidence rather than assess the risk of bias or quality of individual studies. To achieve our purpose, we will construct a descriptive evidence map organized according to four themes that will include: (1) exercise modality (e.g., aerobic, resistance, combined, low-intensity), (2) T cell subtype (e.g., CD4^+^, CD8^+^, regulatory, γδ T cells), (3) age (e.g., children under 18 years, young adults aged 18–40 years, adults aged 40–60 years, and older adults aged 60+ years), and (4) cancer type. Depending on resultant data numbers within each theme, we will present a tabular summary of key study design features and mechanistic/functional endpoints (e.g., metabolic assays, cytokine readouts, omics analysis). Narrative thematic grouping (for example, mitochondrial adaptations versus nutrient-transporter regulation) will also accompany these tables to highlight evidence clusters and gaps, thereby enhancing reproducibility and interpretability.

### Ethical considerations

Ethics approval is not required for this review, as the data will be obtained from existing published literature.

### Analysis and data extraction

Data extraction will be conducted by two independent reviewers using a tailored data extraction form adapted from the JBI template (Aromataris E et al., 2024) (*Multimedia* Figure 2). The form will undergo a pilot phase, applied to the first 10% of the full-text studies included in the review. After this initial trial, the tool will be assessed and refined as needed to ensure all pertinent data are collected. If revisions are made, the final version of the tool will be applied to all full-text studies, including those used in the pilot phase.

### Timeline

Immediately upon peer-review and publication, the final search will be conducted by an experienced academic librarian specializing in health sciences (see Supplementary Appendix SA1 for sample search strategy). Reference screening will then begin and is anticipated to last for 4 months. Data extraction and synthesis will then follow (2 months) with manuscript writing and submission for publication thereafter (2 months).

## Results

This scoping review is expected to produce results towards the end of 2025 or early 2026. The results will be used to inform future studies and grant applications within our working group and will be summated and submitted for publication in a peer-reviewed journal.

## Discussion and expected impact

So far as we can determine, this is the first scoping review to examine how exercise impacts T cell function and metabolism in cancer.

### From cell to society

Understanding how exercise influences the immune system at the cellular level offers several significant advantages for society, particularly in the context of cancer treatment. One of the most important exercise-induced benefits is the stimulation of the immune system, which plays a crucial role in mediating anti-cancer effects. By improving the body’s immune response, exercise may potentiate the activity of immune cells, including T cells, that play a critical role in recognizing and eliminating cancerous cells. Furthermore, exercise might have the potential to enhance the effectiveness of immunotherapies, which have become a cornerstone in cancer treatment. Exercise interventions prior to T cell extraction for ACT could help yield a more functional immune cell used for product manufacturing.

Undoubtedly, exercise can profoundly improve outcomes for cancer patients, yet the underlying reasons are less well characterized. Emerging evidence indicates that exercise can remodel the TME by lowering immunosuppressive signals and promoting the infiltration and effector functions of anti-tumour immune cells. By understanding these mechanisms at a cellular level, researchers and clinicians can prescribe exercise not merely as a general lifestyle recommendation but as an evidence-based adjuvant therapy. This knowledge will also guide the development of combination strategies that integrate structured exercise programs with immunotherapies for better patient outcomes.

Lastly, exercise’s ability to improve overall immune function could extend beyond cancer treatment, fostering general health and resilience in patients undergoing aggressive therapies like chemotherapy and radiotherapy. It can help mitigate some of the adverse side effects of these treatments, such as immune suppression and fatigue, leading to improved quality of life during treatment. Incorporating exercise as an adjunct to cancer therapies, particularly immunotherapy, could thus represent a highly accessible, cost-effective strategy to boost immune response and enhance therapeutic outcomes.

### Strengths and limitations

A key strength of this scoping review is its novelty as well as its comprehensive and systematic approach to mapping the existing literature on the effects of exercise on T cell function and metabolism in the context of cancer. By incorporating both human and *in vivo* animal studies, the review will encompass a broad spectrum of evidence across preclinical and clinical settings. The use of established methodological frameworks, such as the JBI guidance and PRISMA-ScR reporting standards, enhances the transparency and reproducibility of the review process. However, some limitations should be acknowledged. As a scoping review, this study will not include a formal quality assessment or risk of bias appraisal, which may impact the interpretability of the findings. Additionally, restricting inclusion to English-language publications may introduce language bias and limit the comprehensiveness of the evidence base. Finally, the heterogeneity of terms used for our outcome measures across studies may pose challenges to a complete, comprehensive search, and there is a possibility that some studies may be missed in our search.

## Conclusion

In conclusion, this proposed scoping review holds profound implications not only for individual cancer treatment outcomes but also for broader societal health. By enhancing immune system health through exercise, we can improve the efficacy of immunotherapies, benefiting patients on a clinical level. Furthermore, promoting exercise as a complementary strategy could help reduce the healthcare burden by improving overall cancer care, quality of life, and long-term survivorship. Ultimately, bridging the gap between cellular mechanisms and population health offers a promising avenue for advancing cancer treatment and fostering a healthier, more resilient society.

## Data Availability

The original contributions presented in the study are included in the article/Supplementary Material, further inquiries can be directed to the corresponding author.
