# Supplementary material for: Exploring the effects of exercise on T cell function and metabolism in cancer: a scoping review protocol
Source: Front Physiol. 2025 Aug 29;16:1655306. doi: 10.3389/fphys.2025.1655306 (PMC12426026; doi:10.3389/fphys.2025.1655306)
Supplement: Supplementary file 1 [file DataSheet1.pdf]

APPENDIX
Search Strategy

Database: Ovid MEDLINE

Ovid MEDLINE(R) ALL

1 exp Neoplasms/

2 (cancer* or anticancer* or neoplas* or carcinoma* or malignan* or sarcoma* or osteosarcoma* or chondrosarcoma* or melanoma* or lymphoma* or leukemia* or leukaemia* or myeloma* or glioma* or neuroblastoma* or retinoblastoma* or mesothelioma*).tw,kf.

3 1 or 2 [CANCER]

4 exp Exercise/ or exp Exercise Therapy/ or exp Sports/ or exp Physical Endurance/ or Physical Exertion/ or exp Muscle Strength/ or exp Athletes/

5 (exercis* or (train* adj1 (resistance or endurance or strength or weight or interval or cross or cardio* or aerobic or anerobic or plyometric or isometric or eccentric or sprint or jump or step or intensit*)) or physical activit* or physical conditioning or physical training or sport or sports or weightlift* or swim* or bicycl* or walk* or treadmill* or yoga or pilates or tai chi or tai ji or martial art or martial arts or exergam* or exer gam* or hiit or progressive effort* or wheel running or athlet*).tw,kf.

6 exercis*.jw.

7 4 or 5 or 6 [EXERCISE]

8 exp T-Lymphocytes/

9 (t lymphocyte* or t cell or t cells or t regulatory or t helper* or natural killer* nk cell or nk cells or treg or tregs or cd3* or cd4* or cd8*).tw,kf.

10 8 or 9 [T-CELLS]

11 3 and 7 and 10 [CANCER + EXERCISE + T-CELLS]

12 11 not (Comment/ or Editorial/ or Letter/)

13 limit 12 to english
